# Use of ethnic spices by adults in the United States: An exploratory study

**DOI:** 10.15171/hpp.2018.04

**Published:** 2018-01-07

**Authors:** Jonathan Isbill, Jayanthi Kandiah, Jagdish Khubchandani

**Affiliations:** ^1^Honors College, Ball State University, Muncie, IN, USA; ^2^College of Health,Ball State University, Muncie, IN, USA

**Keywords:** Consumer Behavior, Spices, Complementary Therapies, Diet

## Abstract

**Background:** The use of complementary and alternative medicine (CAM) therapies has increased in the United States, but little is known about consumers’ perceptions of use of such therapies. The purpose of this study was to assess knowledge, perceptions, and predictors of spice use for health promotion among adults in the Midwestern US.

**Methods:** UUsing a cross-sectional study design, adults in the Midwestern US (n = 703) completed a valid and reliable survey which was pilot tested with a small convenience sample of adults (n = 38). The study variables included demographic profile, spice use behavior, perceptions about efficacy of spices, and willingness to use spices. Data were analyzed using SPSS to compute descriptive (e.g. percent and frequencies) and inferential statistics (i.e. logistic regression analyses).

**Results:** Almost half of the participants were interested in learning about health benefits of spices (48%), indicated friends and family members as sources of information on spices (50%),and were willing to use spices as CAM therapies (51%). Most (>50%) of the participants were familiar with or had used eight out of the 10 listed spices. The majority of participants (54%)were currently using one or more spices on a daily basis and believed that ginger (64%), garlic(58%), and cinnamon (56%) could promote good health and wellness. In logistic regression analysis, age, gender (odds ratios [OR] = 1.44 and OR = 1.56), income (OR = 1.77), health status(OR = 2.01), and recommendations from healthcare providers (OR = 5.31 and OR = 3.96) were significant predictors of current spice use and willingness to use spices.

**Conclusion:** Individuals in our study did not use many ethnic spices and were unaware of potential health benefits of spices. Greater awareness of ethnic spices for disease prevention and health promotion are needed in this population.

## Introduction

Globally, spices are a group of botanicals known best for their role in cooking and are frequently employed as dietary supplements to enhance health and wellness.^1^ Many of these spices are used in disease management within the field of complementary and alternative medicine (CAM).^1-3^ Preclinical and clinical research results have suggested a strong correlation between usage of certain ethnic spices and positive health benefits against several common illnesses and diseases while reducing health risks, improving metabolism, and providing relief from numerous disease related symptoms.^2-5^ Spices and herbs have been found to possess anti-diabetic, anti-lithogenic, anti-mutagenic, anti-inflammatory, antiviral, antibacterial, as well as other positive health benefits such as reduction of body fat, reduced sodium intake, and enhancing metabolism.^3-9^

Spices have been used for various purposes and explored for their utility for hundreds of years in various parts of the world, but the interest in spices has grown in the Western world only during recent times.^9-12^ The United States Department of Agriculture reports that the consumption of spices in the United States has climbed exponentially over the course of the last half-century, with spices such as ginger and chili pepper being used more frequently than ever before.^10-12^ In the United States, health promotion through CAM is also of growing interest.^12-14^ It has been suggested that more than one-third of all adults currently use some form of CAM. According to the US National Health and Nutrition Examination Survey (NHANES), 5%-10% of adults in the United States utilize botanical supplements such as spices, for health benefits.^13-15^ In addition to the adult population use, spices have also been explored in pediatric populations. For example, more than a tenth of the infants and children have used spices, primarily to remedy minor ailments such as fussiness or stomach complications, and cough and cold. Such increased usage could in part be due to lack of side effects from spices, greater availability than traditional medicines, and consideration of known health benefits of spices.^12-16^

As more Americans are considering the use of spices and herbs for medicinal and therapeutic use, especially to remedy various chronic conditions, reduce disease symptoms, and aid in treatment and management of common health problems, it is prudent to examine consumers’ perceptions about spices and their utility and predictors of spice usage.^12-16^ Although a handful of studies in the United States have observed the use of herbs and spices, they have several limitations.^17-21^ First, the studies are from small convenience samples of individuals. Second, most studies use few itemized questions to examine spice usage without estimating perceptions and predictors of spice usage. Third, studies frequently inquire about CAM usage and do not examine usage of various CAM products such as spices. In addition, the few studies that we found in a comprehensive literature review focused on special groups in the United States such as registered dietitians and dietetics students. Finally, none of the studies looked at general adult population in the Midwestern US. Therefore, the purpose of this study was to assess knowledge, perceptions, and predictors of spice use for health promotion among adults in the Midwestern US.

## Methods

### Sample population and procedures

A snowball sampling procedure was utilized to recruit a convenience sample of adults in the Midwestern US. The sample was invited to take the valid and reliable survey created for this study. An a-priori power analysis was conducted to determine the sample size, we found that with a conservative 95% confidence level, and 5% margin of error, 354 adult participants would be needed to make inferences to the total population of adults in the Midwestern US (approximately, 45 million adults).^22,23^

The inclusion criteria were that participants had to be at least 18 years of age, English speaking, and living in the Midwestern US. A recruiting e-mail communication informed participants about the study purpose and overview of the research and approximate time commitment for completion of the survey. Participants in the community and in social networks were sent a link with the informed consent information and the study survey. Participants were also asked to share the survey link with friends, family members, and community acquaintances in the Midwestern US. Preceding the survey distribution, the study protocol was approved by the Institutional Review Board for research involving human subjects.

### Instrument and measures

Based on a comprehensive literature review and available questionnaires, a 39-item survey was designed to ensure face validity. The survey was reviewed by experts (n = 5) on survey design and consumer sciences to ensure content validity.^22,23^Based on expert suggestions, changes in format and content were included in the final survey. In addition to the demographic and background information items on the survey, a major component of the study survey was the multi-item Consumers Usage of Ethnic Spices Survey (CUES). The CUES was developed and adapted based on revision of a previous survey that explored the familiarity of spices in registered dietitians and dietetic students.^18-20^ CUES has three subscales and the subscale reliabilities were assessed from the final sample of study participants. The first subscale assessed individuals’ usage and familiarity with spices (10 item scale, internal reliability from final sample of respondents α = 0.81). The second subscale assessed individuals’ perceptions about various spices in promoting good health and wellness (10 item scale, internal reliability from final sample of respondents α = 0.90). The final subscale of CUES assessed consumers’ perceptions of the efficacy of various spices in preventing 12 disease types (internal reliability from final sample of respondents α = 0.63).

Stability-reliability of the survey instrument items was assessed by collecting data from a convenience sample of adults in the Midwestern US. A random-sample of 50 adults was invited to participate in reliability testing. These individuals were asked to complete the survey twice within a period of 7 days. Kappa statistics (percentage agreement = ĸ) were used for items on the subscales as they were all categorical variables.^22,23^ A total of 38 completed matched pairs of surveys from individuals were used for reliability testing. The stability reliability coefficients for the various CUES subscale items were found to be acceptable and ranged from 0.63 to 0.90.

### Data analysis

Survey data were entered into the computer and analyzed using the IBM SPSS Statistics for Windows statistical package (Chicago, version 24). Descriptive statistics (frequencies and percentages) were used to describe the participants in terms of their demographic and background characteristics and responses to the questions about usage of spices and perceived efficacy of spice use. To assess participants’ current usage of spices and willingness to use spices in the future, we conducted univariate logistic regression analysis to compute odds of spice usage in various groups (with 95% confidence intervals). The outcomes were categorized as binary variables (yes vs. no) for current daily use of spices and intent to use spices in future and demographic characteristics served as independent predictor variables. Statistical significance was established *a-prior* at *P *≤ 0.05.

## Results

### Background characteristics and spice usage

A final sample of 703 eligible individuals completed the survey (this response was almost twice the number required in power analysis, i.e. n = 354). The majority of the respondents were females (77%), Whites (88%), above the age of 25 years (52%), and earned more than $35 000 per year (Table 1). In relation to health status, majority of the respondents were in excellent or very good health status (62%) and satisfied with current health status (66%). Majority of the participants cooked at home 4 or more times and went out to eat less than 3 times per week (74% and 81% respectively). Almost half of the participants indicated friends and family members as sources of information on spices (50%), were interested in learning about health benefits of spices (48%), and were willing to use spices as CAM treatment (51%). Participants were asked about their familiarity with and usage of spices (Table 2). Majority of the participants were familiar with or had used 8 out of the 10 listed spices. Black pepper and garlic were the 2 most commonly used spices on a daily basis. The majority of participants (n = 380, 54%) were currently using one or more spices on a daily basis. Participants had the least familiarity and usage of curry leaves (79%) and fenugreek (59%).

### Perceived efficacy of spices

Participants were asked about their opinion of the effectiveness of spices in promoting health and wellness and the majority believed that ginger (64%), garlic (58%), and cinnamon (56%) could promote good health and wellness. Participants were least likely to believe that curry leaves (15%) and fenugreek (11%) could be effective in health promotion (Table 3). Additionally, study participants were given specific disease options and asked about the efficacy of various spices in prevention or reduction of disease conditions (Table 4). The vast majority of the participants did not believe that fenugreek (53%), curry leaves (52%), or cilantro (50%) had any role in preventing or reducing specific diseases. On the contrary, majority of the participants listed 7 out of 10 spices as effective in preventing a specific disease with ginger (72%), garlic (68%), and cinnamon (67%) listed as effective by more than two-thirds of the participants.

### Predictors of spice usage

Several demographic and health related variables were significant predictors of current spice use and willingness to use spices in the future (Table 5). Females (odds ratio [OR] = 1.44, 95% CI = 1.26-2.80), those who were older than 55 years (OR = 1.92, 95% CI = 1.47-2.45) individuals with annual income more than $75 000 (OR = 1.71, 95%CI = 1.10-2.25), graduate degree holders (OR = 1.27, 95% CI = 1.10-1.49), those who had ever met a registered dietitian (OR = 4.05, 95% CI = 2.90-6.03), and those who were encouraged by a health professional to consume spices (OR = 5.31, 95% CI = 3.06-7.02) were statistically significantly more likely to report usage of spices on a daily basis compared to their counterparts. Those who ate out of home more and did not have interest in learning about health benefits of spices were statistically significantly less likely to currently use spices on a daily basis (Table 5).

In relation to future spice use, females (OR = 1.56, 95% CI = 1.17-2.25), individuals older than 55 years (OR = 1.89, 95% CI = 1.36-2.16), those with a graduate degree (OR = 1.25, 95% CI = 1.06-1.44), individuals reporting current health status as fair or poor (OR = 2.01, 95% CI = 1.17-3.80), those who were dissatisfied with current health status (OR = 3.00, 95% CI = 2.07-4.05), those who cooked at home four or more times (OR = 1.70, 95% CI = 1.26-2.34), those with an interest in knowing about health benefits of spices (OR = 6.30, 95% CI = 4.14-8.01), individuals who had ever met a registered dietitian (OR = 3.60, 95% CI = 2.01-4.05), and those who were ever encouraged by a health professional to consume spices (OR = 3.96, 95% CI = 2.07-4.41) were statistically significantly more likely to be willing to use spices in the future compared to their counterparts. However, individuals who were eating out of home more than four times a week and those who were disinterested in knowing about health benefits of spices were less likely to be willing to use spices in the future.

## Discussion

Ethnic spices have long been studied in various regions around the world as medicinal agents, for culinary use, and as health promotion supplements.^1-3^ Yet there exists a lack of understanding about usage and perceptions of common ethnic spices in the United States.^17-21^ With increasing prevalence of spice usage in the United States as a form of CAM, this study explored consumers’ perceptions and predictors of spice usage in a unique population (adults in the Midwestern US). Several unique findings of this study warrant attention. Majority of the participants reported using a spice on a daily basis, were interested in learning more about the health benefits of spices, and were willing to use spices as CAM. This is interesting given that our sample mostly consisted of people from Midwestern US and may not have the exposure to or cultural diversity in available food items. Along the Western and Eastern regions of the United States, there is greater ethnic and cultural diversity when compared to the Midwestern regions. Furthermore, those with higher income and more education levels had a greater propensity to use spices, which speaks of awareness and affordability that might be low in the Midwestern US. Most of the participants were familiar with and believed in the efficacy of few spices in health promotion and disease prevention. The common spices in this category were garlic, cinnamon, ginger, and chili pepper. This finding is in agreement with studies of US based registered dietitians and dietetic students who reported greater benefits of and familiarity with these few spices, especially as it relates to prevention and reduction of diabetes, cardiovascular disease, and gastrointestinal diseases.^18-20^ In addition, these are widely known spices as there is ample evidence from studies to show the utility and health benefits of these commonly used spices.^24-26^ Participants were least likely to use or be familiar with fenugreek and curry leaves, which are highly utilized in many Asian and Middle-Eastern cuisines, these cuisines may not be as common in the Midwestern US. This lack of awareness about many spices could also be in part because less than a fifth of study participants saw a registered dietitian or received advice to consume spices from a health professional, highlighting the role of such professionals in advancing the use of CAM approaches.^18-20^ Results of this study also revealed that consumers received most of their information about spices from friends, family, and the internet, sources that may not be credible.

The most interesting and unique finding of this study is the predictors of current usage and future use willingness. Females, more educated, older, those who make higher incomes, and individuals who cook at home more often were significantly more likely to use or willing to use spices. This is logical as women are commonly responsible as primary providers of meals for family and may have better knowledge, those with higher incomes may be more able to afford spices, and those with higher education could possibly know more about health benefits of spices. However, some predictors of spice usage need additional research. For example, those with poor health status were less likely to report current usage of spices, but two times more likely to be willing to use spices compared to those with good health. It could be possible that those currently using spices are in good health and those who have poor health may be willing to use spices for health benefits. There is evidence in published literature to show that individuals with severe chronic illness might be willing to participate in research, experiment with therapies, and look for any possible solutions in the hope of finding treatment of existing disease conditions.^13,21,27^ This can also be related to another finding of our study where those who are dissatisfied with their health are significantly more likely to be willing to consume spices in the future. Given the rising prevalence of chronic diseases in US population and the fact that most individuals who have a chronic disease often have multiple health issues, spices can certainly play a major role in preventing diseases and promoting good health as a CAM approach.^13-16,24-26^ According to a study conducted with Oregonian dietitians, 80% of them believed that food supplements can play an effective role in preventing and treating chronic diseases; dietitians can therefore, serve as advocates for use of spices as a CAM approach along with other supplements.^28^ Our study has profound implications for future research and health promotion practice. Overall, as majority of participants shared interest in learning about the health benefits of spices, there are avenues for providing knowledge. Evidence-based strategies and resources could be integrated in both culinary, nutrition and allied health courses throughout all phases of educational advancement (e.g. high school, university, etc). Additionally, community garden initiatives, local farmers, and health care professionals could also provide educational opportunities to increase consumers’ awareness of the health benefits of spices and utility as CAM.^17-21^ While reasonable evidence exists for the efficacy of spices, further research is needed on the various benefits of spices- such studies should be conducted in larger community and clinic based samples. Some spices could more likely be used or known to consumers because of widespread and consistent evidence about their efficacy in disease prevention and health promotion. Registered dietitians and nutritionists have a key role in research and education related to spices and their uses.^28-30^ In addition, they can serve as credible resource persons for information and prescription. In fact, a study of Californian dietitians, most of the dietitians (greater than two-thirds) believed that a dietitian should be proficient in discussing about dietary supplements with clients and be able to inform the public.^29^ Studies have shown that more Americans suffer from chronic conditions now. As many ethnic spices have potential to aid in not just one, but also multiple chronic disease treatments, the use of ethnic spices could help prevent many co-morbidities simultaneously without precipitation of any side effects.

The results of our study should be viewed in light of several potential limitations.^22,23^ First, the results may be impacted by all the traditional limitations of survey research (i.e., self-reported data, selection bias, recall bias, and inability to establish cause and effect relationships). In addition, participants could have given socially desirable responses to study questions. The questionnaire was monothematic in nature and mostly closed format in nature, this could be a threat to internal validity. Our sample was limited to adults in the Midwestern US and cannot be generalized to adults across the United States. However, in spite of these factors, we observed high reliability and validity of the instrument in our unique sample, have the largest sample report to date for general population in the United States, and we also explored various predictors of spice usage, making our study unique.

## Conclusion

This study was an attempt to assess perceptions about and the predictors of spice usage in a sample of adults in the Midwestern US. Individuals in our study did not use many ethnic spices and were unaware of potential health benefits of spices. Greater awareness and marketing of such ethnic spices as complementary and alternative options for disease prevention and health promotion are needed in this population. Given the spice use predictors we found, the information can be used to reach out to individuals who are less likely to use spices and may find it beneficial to use spices for health promotion.

## Ethical approval

The study protocol and procedures were approved by the Institutional Review Board for Human Subjects Research at Ball State University, USA.

## Competing interests

We declare no conflicts of interest.

## Authors’ contributions

JI and JayK contributed to the conception and design of the study and the writing and editing of the manuscript. JK analyzed and interpreted the data and wrote the results. JayK and JK provided critical revision of the article. JI, JayK, and JK have approved this submission. All authors contributed equally to the work of the manuscript.

## Funding

The authors did not receive any funding for this study.

**Table 1:** Demographic and background characteristics of participants

**Variable**	**No. (%)**
Gender	
Male	161 (23)
Female	542 (77)
Race	
White	626 (88)
Non-White	77 (12)
Age (y)	
18-24	331 (47)
25-54	266 (37)
≥ 55	108 (15)
Annual income ($)	
≤34 999	197 (28)
35 000-74 999	249 (34)
≥75 000	254 (35)
Employment status	
Full time	297 (42)
Part time	183 (26)
Unemployed	223 (32)
Education	
≤High school graduate	189 (27)
<Bachelors	169 (24)
≥Bachelors	148 (21)
≥Graduate degree	188 (27)
Current health status	
Excellent/ Very good	433 (62)
Good	220 (31)
≥Fair/ Poor	48 (7)
Satisfaction with current health status	
Very satisfied/satisfied/somewhat satisfied	463 (66)
Neutral	65 (9)
Very dissatisfied/dissatisfied/ somewhat dissatisfied	175 (25)
Cooking at home	
<1 time per week	14 (2)
1-3 times per week	168 (24)
≥4 times per week	520 (74)
Eating out of home	
<1 time per week	160 (23)
1-3 times per week	406 (58)
≥4 times per week	136 (19)
Source of information about spices	
Friends/ Family	352 (50)
Internet	309 (44)
Magazines	139 (20)
Television	102 (15)
Physician	70 (10)
Registered Dietitian	44 (6)
Other	94 (13)
Interest in learning about health benefits of spices	
Very interested/interested	339 (48)
Neutral	106 (15)
Very disinterested/disinterested	251 (36)
Willingness to use spices as CAM
Very willing/willing	360 (51)
Neutral	106 (15)
Very unwilling/unwilling	230 (33)
Ever met with a registered dietitian	136 (19)
Ever encouraged by a health professional to consume spices	100 (14)

N = 703 (numbers may not add up to 100% due to missing values or rounding off).

**Table 2:** Spice use and familiarity in study participants

**Spice**	**No. (%)**
Black Pepper	
Use daily	400 (57)
Know about/ Used at least once	282 (40)
Never used/ Don’t know about	17 (2)
Garlic	
Use daily	288 (41)
Know about/ Used at least once	397 (56)
Never used/ Don’t know about	9 (1)
Cinnamon	
Use daily	106 (15)
Know about/ used at least once	578 (82)
Never used/ Don’t know about	24 (3)
Chili Pepper	
Use daily	83 (12)
Know about/ Used at least once	601 (85)
Never used/ Don’t know about	16 (2)
Cilantro	
Use daily	57 (8)
Know about/ Used at least once	617 (88)
Never used/ Don’t know about	12 (1)
Ginger	
Use daily	55 (8)
Know about/ Used at least once	627 (89)
Never used/ Don’t know about	15 (2)
Turmeric	
Use daily	38 (5)
Know about/ Used at least once	569 (81)
Never used/ Don’t know about	96 (14)
Cloves	
Use daily	14 (2)
Know about/ Used at least once	650 (92)
Never used/ Don’t know about	41 (6)
Curry leaves	
Use daily	8 (1)
Know about/ Used at least once	140 (20)
Never used/ Don’t know about	546 (78)
Fenugreek	
Use daily	7 (1)
Know about/ Used at least once	280 (40)
Never used/ Don’t know about	414 (59)

N = 703 (numbers may not add up to 100% due to missing values or rounding off).

**Table 3:** Perceived efficacy of spices in promoting health and wellness

**Spice**	**Effective n (% Agreement)**
Ginger	450 (64)
Garlic	404 (58)
Cinnamon	392 (56)
Chili pepper	300 (43)
Turmeric	251 (36)
Cilantro	168 (24)
Cloves	163 (23)
Black pepper	163 (23)
Curry leaves	108 (15)
Fenugreek	76 (11)

N = 703 (% = indicates individuals who believe that a certain spice can promote health and wellness).

**Table 4:** Consumer perception of efficacy of spices in preventing/reducing diseases

**Spice**	**None %**	**Cancer %**	**Cold/Flu %**	**CVD %**	**Diabetes %**	**GI Dis %**	**HIV/AIDS %**	**MSD %**	**Obesity %**	**Oral %**	**ND/ MI %**	**Kid Dis %**	**Res Dis %**
Ginger	28	12	23	13	9	31	5	13	11	12	9	9	12
Garlic	32	11	24	20	11	9	5	9	11	9	8	8	11
Cinnamon	33	10	20	15	20	12	5	9	17	14	12	9	10
Chili pepper	38	8	18	12	6	8	3	12	16	4	6	6	11
Turmeric	41	15	10	13	9	10	5	16	12	7	9	8	8
Cloves	45	5	11	8	6	9	3	7	7	14	5	6	10
Black pepper	49	4	11	8	5	7	2	7	8	3	7	5	8
Cilantro	50	4	8	7	5	9	2	6	8	8	5	6	7
Curry Leaves	52	7	7	8	5	8	4	5	8	5	7	6	7
Fenugreek	53	7	5	6	6	6	3	6	7	5	5	6	4

N = 703 (% = indicates individuals who believe that a certain spice can prevent or reduce diseases listed in table columns). Abbreviation: CVD= Cardiovascular disease; GI Dis= Gastrointestinal diseases; MSD= Musculoskeletal disease; ND/MI= Neurological dysfunction/mental disorders Kid Dis= Kidney diseases; Res Dis= Respiratory diseases; None= indicates that participants believed a certain spice has no use in preventing disease.

**Table 5:** Predictors of current use or future use of spices

**Variable**	**OR (95% CI) Current Use**	**OR (95% CI) Future Use**
Gender		
Male	1 (Ref)	1 (Ref)
Female	1.44 (1.26-2.80)*	1.56 (1.17-2.25)*
Race		
White	1 (Ref)	1 (Ref)
Non-White	1.17 (0.98-1.24)	1.35 (0.81-1.71)
Age (y)		
18-24	1 (Ref)	1 (Ref)
25-54	1.80 (1.35-2.34)*	1.32 (0.96-1.80)
≥55	1.92 (1.47-2.45)*	1.89 (1.36-2.16)*
Annual income ($)		
≤34 999	1 (Ref)	1 (Ref)
35 000-74 999	1.27 (0.98-1.44)	1.02 (0.72-1.18)
≥75 000	1.71 (1.10-2.25)*	1.80 (0.93-2.01)
Employment Status		
Full time	1.22 (0.95-1.76)	1.17 (0.90-1.71)
Part time	1.13 (0.86-1.68)	1.08 (0.81-1.62)
Unemployed	1 (Ref)	1 (Ref)
Education		
≤High school graduate	1 (Ref)	1 (Ref)
<Bachelors	1.10 (0.81-1.35)	1.03 (0.72-1.21)
≥Bachelors	1.17 (0.89-1.40)	1.11 (0.81-1.35)
≥Graduate degree	1.27 (1.10-1.49)*	1.25 (1.06-1.44)*
Current health status		
Good	1 (Ref)	1 (Ref)
Excellent/ very good	1.44 (0.90-1.99)	1.53 (0.93-2.01)
≥Fair/poor	0.98 (0.81-1.17)	2.01 (1.17-3.80)*
Satisfaction with current health status		
Neutral	1 (Ref)	1 (Ref)
Very dissatisfied/dissatisfied/ somewhat dissatisfied	0.90 (0.72-1.08)	3.00 (2.07-4.05)*
Very satisfied/satisfied/ somewhat satisfied	1.37 (0.89-1.79)	2.70 (1.80-3.33)*
Cooking at home		
<1 time per week	1 (Ref)	1 (Ref)
1-3 times per week	1.22 (0.98-1.53)	1.17 (0.93-1.30)
≥ 4 times per week	1.89 (1.35-2.43)*	1.70 (1.26-2.34)*
Eating out of home		
< 1 time per week	1 (Ref)	1 (Ref)
1-3 times per week	0.79 (0.57-0.88)*	0.81 (0.63-1.26)
≥ 4 times per week	0.69 (0.54-0.76)*	0.72 (0.54-0.93)*
Interest in learning about health benefits of spices		
Neutral	1 (Ref)	1 (Ref)
Very disinterested/disinterested	0.63 (0.54-0.81)*	0.72 (0.44-1.02)
Very interested/interested	2.29 (1.89-2.87)*	6.30 (4.14-8.01)*
Ever met with a registered dietitian (Yes vs. No)	4.05 (2.90-6.03)*	3.60 (2.01-4.05)*
Ever encouraged by a health professional to consume spices (Yes vs. No)	5.31 (3.06-7.02)*	3.96 (2.07-4.41)*

N = 703, * indicates *P* < 0.05; Ref = indicates the reference or comparison group. OR= Odds ratios indicating probability of an outcome (i.e. current use or future use); 95% CI = confidence intervals for odds; Current use = individuals who use any spice on daily basis; Future use = individuals willing to consume spices as CAM in the future.
